# Exploring gaps, strategies and solutions for primary care research mentorship in the African context: A workshop report

**DOI:** 10.4102/phcfm.v12i1.2320

**Published:** 2020-05-25

**Authors:** Chelsea M. McGuire, Kenneth Yakubu, Nana K. Ayisi-Boateng, Keneilwe Motlhatlhedi, Pius Ameh, Bola B. Fatusin, Martha Makwero, Louis S. Jenkins

**Affiliations:** 1 Family Medicine Speciality Training Program, Lesotho-Boston Health Alliance, Leribe, Lesotho; 2 Department of Family Medicine, Boston University School of Medicine, Boston, United States; 3 Faculty of Medicine, University of New South Wales, Sydney, Australia; 4 The George Institute for Global Health, Sydney, Australia; 5 Department of Family Medicine, University of Jos and Jos University Teaching Hospital, Jos, Nigeria; 6 School of Medicine and Dentistry, Kwame Nkrumah University of Science and Technology, Kumasi, Ghana; 7 Department of Family Medicine and Public Health, Faculty of Medicine, University of Botswana, Gaborone, Botswana; 8 Department of Family Medicine, Federal Medical Centre, Keffi, Nigeria; 9 Consultant Family Physician and Academic Coordinator, Family Medicine Department, Federal Medical Centre, Gusau, Nigeria; 10 Department of Family Medicine, School of Public Health and Family Medicine, University of Malawi, Blantyre, Malawi; 11 Department of Family and Emergency Medicine, Faculty of Health Sciences, Stellenbosch University, Stellenbosch, South Africa; 12 Department of Family and Emergency Medicine, George Hospital, Western Cape Department of Health, George, South Africa

**Keywords:** research, mentorship, primary care, general practice, family medicine, sub-Saharan Africa, workshop report

## Abstract

**Background:**

Primary care needs research to generate evidence relevant to community needs; however, there is a lack of research engagement among primary care physicians, especially in sub-Saharan Africa. Improved research mentorship for family physicians (FPs) can help address prevailing knowledge and practice gaps in primary care research.

**Workshop process:**

During the 6th annual Africa Regional Conference of the World Organization of Family Doctors (WONCA), we conducted three workshops on research mentorship for African FPs. Two workshops (one online and one onsite at the pre-conference) were geared towards the young doctors’ movement of WONCA Africa. The third was onsite during the main conference. Following a brief presentation on the concept of research mentorship and known gaps, participants broke into small groups and discussed additional gaps, solutions and anticipated readiness for implementing these solutions. We used a content analysis to summarise key concepts and had participants to review the findings.

**Workshop findings:**

Identified gaps related to mentees’ difficulty initiating and maintaining mentorship relationships and an overall shortage of capable and willing mentors. Organisational solutions focused on capacity building and creating a culture of mentorship. Interpersonal solutions focused on reducing the power distance and increasing reflectivity and feedback. Increasing the use of research networks and both peer and online mentorship were advocated. Barriers to readiness included resource constraints and competing priorities.

**Conclusion:**

A multi-level approach is needed to address the gaps in research mentorship for African FPs. Identified solutions hold potential for supporting the research engagement needed to improve the population health across Africa.

## Introduction

In June 2019, over 180 family medicine and general practice trainees, clinicians, educators and researchers convened in Kampala, Uganda, for the 6th annual Africa Regional Conference of the World Organization of Family Doctors (WONCA). It provided an opportunity to discuss the status of family medicine around the continent, share best practices to promote its development and tackle some of the most pressing challenges facing primary care in Africa. Among these challenges is a gap in primary care research, especially in sub-Saharan Africa.^1^ Mentorship is a crucial driver of research engagement.^2,3^ Thus, a series of workshops were held to advance the issue of research mentorship for family physicians (FPs) in the African context. This report summarises the workshop results, focusing on the innovative solutions that were generated with the hope of encouraging their implementation to build sustainable research mentorship for FPs in Africa.

## Workshop process

We conducted three workshops on ‘Innovative Approaches to Research Mentorship in Primary Care: Exploring Gaps, Strategies and Solutions’. The first workshop was part of the pre-conference for AfriWon Renaissance (AfriWon), the young doctors’ movement (YDM) of WONCA Africa. The second workshop took place during the main conference and was geared towards a wider audience, focusing more on the organisational level. Both workshops opened with a brief presentation which defined research mentorship as: (1) instrumental support regarding the ‘how-to’ of research; (2) connection to research resources such as software, experts in research methodology and funding; (3) research career development support; and (4) psychosocial support to facilitate emotional and personal development.^4^ The presentation also covered known gaps in research mentorship from the literature and the modified Delphi technique structure of the workshop. Participants then broke into small groups of 8–12 people and discussed (1) additional gaps or challenges to research mentorship; (2) context-specific, innovative solutions to these gaps; and (3) anticipated readiness and potential barriers to implementing these solutions in the participants’ home context. Group leaders captured all generated ideas and presented the results in a large group discussion. A single list for each domain was compiled and participants then voted on the ideas. However, because of time limitations, the voting process was not completed for all domains. The third workshop took place virtually using a combination of WhatsApp messenger and Zoom video conferencing. Similar to the first workshop, it was geared towards the AfriWon demographic and intended to follow the same format. However, because of technical and network difficulties, only one breakout session occurred, and there was no final voting session.

RStudio (Version 1.2.1335) statistical software was used to analyse participant demographics. Given the departure from the typical modified Delphi technique voting process, we did not achieve consensus during these workshops. Rather, two team members used a content analysis approach to categorise key concepts from all three workshops under three domains: gaps, solutions and readiness.^5^ These categories were then verified by a third team member and summarised. Preliminary findings were shared with participants for verification and inputs were incorporated.

## Workshop findings

### Participants

The AfriWon pre-conference workshop had 30 attendees, and 26 attendees shared their demographic information. Participants were 46% female, ranged from 4 years pre-qualification to 6 years post-qualification, had a mean age of 36.8 (standard deviation [s.d.] 5.1) and came from 12 countries. The main WONCA workshop had 17 participants, and 16 participants provided demographic information. Participants were 13% female, ranged from first-year medical students to 30 years post-qualification, had a mean age of 38.9 (s.d. 6.2) and came from seven countries. Sign-up for the virtual workshop garnered 26 individuals. However, only eight were able to join the workshop, and no participants filled out the demographic survey. Of the 26 participants who provided a valid email address, nine provided feedback on preliminary workshop findings during the 1-week participant-checking period.

### Gaps

The identified gaps fell into three categories relating to mentees, mentors and institutions. Concerning mentees, participants noted that they have difficulty initiating mentorship relationships, often because of a perceived power difference between themselves and potential mentors. After a relationship is initiated, mentees may not continue to reach out to the mentors because of poor understanding of the mentors’ role. For mentors, attitudes such as paternalism, an unwillingness to ‘truly mentor’ and seeing themselves as supervisors instead of mentors were mentioned. A lack of confidence to mentor was also noted. Institutional gaps included an overall shortage of mentors and lack of a mentorship ‘culture’. Little or no undergraduate exposure to mentorship and insufficient time for mentorship because of work demands were acknowledged. Inadequate funding and lack of institutional support for research inhibit overall research engagement and therefore research mentorship.

### Solutions

The solutions offered also broadly fell into three categories: organisational, interpersonal and specific innovations for mentorship promotion and delivery (Box 1). At the organisational level, attendees emphasised capacity building in research mentorship. Participants suggested the need for explicit research mentorship training for mentors and mentees, starting at the undergraduate level. Participants recommended this training focus on interpersonal communication strategies needed for successful mentorship. Additional training on research-related skills such as time management, research methods, writing and grant applications was also mentioned. To increase the supply of mentors, policies that encourage FPs to pursue PhDs were advocated. Another solution was institution-based policies on research mentorship, including incentives and rewards for mentoring. Finally, participants expressed the need to shift the overall organisational culture, so research is part of everyday conversation and an integral part of family medicine practice. Research should not just be a graduation requirement, but part of daily work to find solutions useful for patients and health systems.

BOX 1 Workshop-generated solutions.

**Organisational**

- Provide explicit research mentorship training (1) for both mentors and mentees, (2) starting at the undergraduate level and (3) focusing on interpersonal communication strategies for effective mentoring.
- Develop policies to (1) increase the supply of mentors by supporting additional FPs to pursue PhDs and (2) outline guidelines to incentivise research mentorship.
- Build research culture within organisations such that it is seen as an integral part of family practice and ensure ongoing research skill development opportunities in areas such as time management, research methods, writing and grant applications.

**Interpersonal**

- Bridge the power distance between mentors and mentees through (1) informal ‘Meet-and-Greet’ sessions to make potential mentors less intimidating to mentees; (2) encouraging mentors to reflect on past challenges that they experienced as mentees; and (3) ensuring feedback goes both ways (e.g. mentees also give feedback to their mentors).
- Promote co-publishing between mentors and mentees.
- Encourage strategic selection of research topics that are exciting, achievable and interesting for both the mentor and mentee.

**Specific innovations**

- Virtual mentorship, either one-on-one or through mentorship groups.
- Use social media to share positive examples of research mentorship for others to emulate.
- Build and support local research networks.
- Peer and ‘near-peer’ mentorship relationships.

Note: Box 1 contains a summary of the proposed solutions to increase research mentorship for African family physicians. This list of solutions was generated by a content analysis of the results from the three workshops.

FP, family physician.

Interpersonal solutions focused on bridging the gap between mentors and mentees. Participants described the need to decrease the power differential within the hierarchy in academia, between faculty and non-faculty members, and medical disciplines in general. Some examples of how to break down these barriers were offered, including informal ‘Meet-and-Greet’ sessions where potential mentors are more approachable to potential mentees. Another solution aimed at encouraging mentors to reflect on their own past challenges as mentees. In addition, mentees were urged to give feedback to their mentors. Encouraging mentors to co-publish with trainees was identified as pertinent. Finally, mentees were advised to be innovative in their choice of research topics, strategically selecting topics that are exciting, achievable and interesting for both themselves and their identified mentor.

A broad range of innovative solutions for research mentorship were explored. One was virtual mentorship. This could be one-on-one or through online mentorship groups. Social media was identified as a useful tool for sharing positive examples of research mentorship for others to emulate. Other ideas included using mentorship teams, expanding local research networks and encouraging peer and ‘near-peer’ mentorship (e.g. between senior and junior registrars or doctorate and master’s students).

### Readiness

Following discussion of solutions, several key barriers to readiness were identified. Participants shared reservations about their institutions’ overall capacity and support for research, which make buy-in and implementation of the potential solutions difficult. The ever-present issue of competing priorities and time constraints for busy FPs, lack of good systems for mentor–mentee matching and the threat of burnout among FPs were noted. A lack of resources relevant to both research engagement and mentorship, such as reliable internet connection, was identified. Finally, gender-related barriers, including gender-based violence, harassment and a shortage of female mentors, were also shared.

## Discussion

The active participation in these workshops demonstrated a recognised need to increase access to quality research mentorship among FPs in Africa. There are challenges, the most significant of which may be time limitations and the hierarchical culture of medicine and research, especially within the African context. Proffered solutions include building capacity for research mentorship and improving interpersonal relations between mentees and mentors through specific training on interpersonal communication. Published research mentorship competencies in this domain include listening deeply, encouraging mentees to speak, communicating with compassion, providing constructive feedback and practising reflexivity.^3^ These strategies can help create the safety needed for research mentorship to thrive.^2^ An observed limitation of the workshop was the prevailing ambiguity between research supervision and mentorship. In the case of trainees, separating these may be needed to create a safe mentorship environment away from evaluation and grading. A YDM meeting report supports our workshop findings on the role of peer, near-peer and e-mentorship to promote research among young FPs.^6^ Successful mentorship programmes must be contextual and recognise diversity. Efforts should work to build simultaneously a culture of research and of mentorship.^1,2^

## Conclusion

The workshop findings suggest that a multi-pronged approach is needed to address the gaps in research mentorship for African FPs, including efforts to (1) extend currently available mentorship through research networks, peer mentorship and e-mentorship; (2) build additional research mentorship capacity by increasing enrolment of African FPs into PhD programmes; (3) provide dedicated mentorship training to both mentors and mentees alike; and (4) support the development of a positive research culture within family medicine through advocacy, increased research funding, protected time and genuine commitment by institutions. These strategies hold potential for catalysing the primary care research engagement needed to improve the health of populations across Africa.
